# Linkage of gut microbiota dysbiosis to chronic kidney disease in patients with graded proteinuria levels

**DOI:** 10.1128/spectrum.03294-25

**Published:** 2026-04-23

**Authors:** Pan Wang, Minjie Wang, Yang Shen, Yaxuan Yao, Kaixin Yan, Siyuan Wang, Zipeng Li, Ying Dong, Bo Li, Jing Li

**Affiliations:** 1 Heart Center and Beijing Key Laboratory of Hypertension, Beijing Chaoyang Hospital, Capital Medical University 74639, Beijing, China; 2 Medical research center, Beijing Chaoyang Hospital, Capital Medical University 74639, Beijing, China; 3 Department of Nephrology, Beijing Chaoyang Hospital, Capital Medical University 74639, Beijing, China; Tongji University, Shanghai, China

**Keywords:** chronic kidney disease, proteinuria, gut microbiota, dysbiosis

## Abstract

Chronic kidney disease (CKD) is highly prevalent worldwide, with proteinuria serving as a key diagnostic and prognostic indicator. The concept of the “gut-kidney axis” suggests that gut microbiota dysbiosis may contribute to CKD pathogenesis; however, its relationship with proteinuria severity remains unclear. This study investigated dynamic changes in gut microbiota composition and function among CKD patients with varying levels of proteinuria, enrolling 148 participants: 54 healthy controls (HC), 49 CKD patients with mild proteinuria (PROU_L), and 45 with massive proteinuria (PROU_M). Fecal samples were analyzed using 16S rRNA gene sequencing to characterize microbiota structure, differential taxa, and functional potential, alongside clinical indicators and Kyoto Encyclopedia of Genes and Genomes (KEGG) pathway analysis. Compared with HC, CKD patients exhibited significantly reduced gut microbiota ɑ-diversity (Chao1 and Shannon indices), which increased as proteinuria worsened. β-diversity also differed significantly among groups. The Faecalibacterium-dominated enterotype (beneficial) declined from 50.0% (HC) to 40.82% (PROU_L) and 28.9% (PROU_M), whereas the Prevotella-dominated enterotype (potentially pathogenic) increased from 7.4% (HC) to 20.41% (PROU_L) and 24.4% (PROU_M). With worsening proteinuria, probiotics, such as Akkermansia and Coprococcus, decreased progressively, while potentially pathogenic bacteria, including Enterobacteriaceae, Haemophilus, and Ruminococcus, were enriched. KEGG-based functional analysis indicated that microbiota nitrogen metabolism became increasingly overactivated from HC to PROU_L and PROU_M, whereas unsaturated fatty acid synthesis was inhibited. Enzymes such as fumarate reductase correlated with the degree of proteinuria. This study characterizes gut microbiota dysbiosis in CKD patients with varying proteinuria levels, revealing reduced probiotics, enrichment of pathogens, and metabolic dysfunction, which may contribute to the progression of renal injury.

**IMPORTANCE:**

Chronic kidney disease (CKD) affects nearly one billion people and remains a leading global cause of death; yet the molecular dialog between gut-microbiome imbalance and the severity of proteinuria is poorly defined. We profiled 148 participants—54 healthy controls, 49 CKD patients with mild proteinuria, and 45 with massive proteinuria—and uncovered a graded dysbiosis that intensifies as proteinuria worsens. Faecalibacterium-dominated enterotypes decline, whereas Prevotella-dominated communities expand; beneficial taxa, such as Akkermansia and Coprococcus, are progressively displaced by uremic-toxin producers (Enterobacteriaceae, Haemophilus). Concurrently, nitrogen metabolism pathways become hyperactive while unsaturated fatty acid synthesis is suppressed. These data illuminate the gut-kidney axis as a driver of CKD progression, deliver stage-specific microbial biomarkers for precision risk stratification, and identify tractable microecological targets for early intervention.

## INTRODUCTION

Chronic kidney disease (CKD) is a heterogeneous condition characterized by a progressive decline in renal function (glomerular filtration rate [eGFR] < 60 mL/min/1.73 m²) or persistent proteinuria for ≥3 months (1). The global prevalence of CKD is estimated at 8%–16%, affecting approximately 850 million individuals worldwide, and CKD has become one of the fastest-growing causes of mortality worldwide (2). Diabetes, hypertension, and glomerulonephritis are common etiologies of CKD. Early symptoms are often insidious, resulting in many patients presenting with advanced renal failure at diagnosis (3). CKD not only increases the risk of end-stage renal disease (ESRD) but is also associated with a high incidence of cardiovascular events and elevated mortality. The annual mortality rate among patients receiving dialysis reaches 10%–20%, with cardiovascular disease accounting for 40%–50% of deaths (4, 5). Global burden of disease studies estimate that CKD causes 1.53 million deaths annually and is projected to become the tenth leading cause of global disease burden by 2050 (5). Consequently, identifying novel therapeutic targets is critical for improving the prognosis of CKD patients.

Proteinuria is a key indicator for the diagnosis and staging of CKD, defined as urinary protein excretion ≥30 mg/24 h or a urinary albumin-to-creatinine ratio ≥30 mg/g (6). According to the KDIGO guidelines, proteinuria severity, together with CKD stage, forms a risk assessment system, and massive proteinuria indicates an extremely high risk of renal disease progression (5). Numerous studies have demonstrated that proteinuria is not only a marker of renal injury but also an independent risk factor for CKD progression. Higher levels of proteinuria are associated with faster declines in eGFR and a markedly increased risk of ESRD (1, 7, 8). For instance, in patients with IgA nephropathy, those with persistent proteinuria >1 g/24 h exhibit an approximately 15% lower 20-year dialysis-free survival rate compared with patients with proteinuria <0.5 g/24 h (9). Proteinuria severity is positively correlated with endothelial dysfunction, cardiovascular events, and all-cause mortality, and it also serves as a key indicator for monitoring treatment response. For example, a reduction in proteinuria by ≥30% following ACEI/ARB therapy indicates a favorable prognostic improvement (8, 10).

The gut microbiota represents the largest microbial ecosystem in the human body, comprising over 100 trillion microbial cells (11). It plays a crucial role in maintaining health by regulating digestion, synthesizing essential vitamins, and preserving intestinal barrier integrity (12). Dysbiosis of the gut microbiota is closely linked to the development and progression of multiple diseases, including obesity, diabetes, inflammatory bowel disease, and cardiovascular disease (13, 14). In recent years, the concept of the “gut-kidney axis” has attracted increasing attention, and a growing body of evidence suggests that gut microbiota dysbiosis may contribute to CKD pathogenesis and progression through mechanisms, such as the production of uremic toxins, impairment of intestinal barrier function, and induction of chronic inflammation (15, 16). The gut microbiota in CKD patients is characterized by a reduction in short-chain fatty acid (SCFA)-producing probiotics (e.g., *Bifidobacterium*, *Lactobacillus*), enrichment of urease- and indole-producing pathogenic bacteria (e.g., *Enterobacteriaceae*, *Clostridium*), and decreased microbial diversity (17, 18). Uremic toxins metabolically produced by the gut microbiota, such as p-cresyl sulfate (PC), indoxyl sulfate (IS), and trimethylamine N-oxide (TMAO), accumulate significantly in CKD patients (15). These toxins are directly associated with declining renal function and increased cardiovascular risk (19, 20). Specifically, TMAO can induce renal oxidative stress and fibrosis via activation of the NADPH oxidase signaling pathway, while IS and PC are closely linked to vascular calcification (21).

Although gut microbiota dysbiosis is closely related to CKD pathogenesis, most studies have focused on advanced or uremic stages of the disease. Research on early-stage CKD, particularly during the proteinuria stage, remains limited. Whether changes in proteinuria levels are associated with gut microbiota dysbiosis is unclear. This study aims to investigate the relationship between gut microbiota dysbiosis and CKD progression by analyzing gut microbiota characteristics in CKD patients with differing proteinuria levels (normal control group, mild proteinuria group, and massive proteinuria group), thereby providing new insights and potential targets for early CKD intervention.

## MATERIALS AND METHODS

### Study population

In this cross-sectional observational study, 148 subjects were enrolled from the Department of Nephrology, Beijing Chaoyang Hospital, Capital Medical University. The cohort comprised 54 HC and 94 patients with CKD. Exclusion criteria were as follows: pregnancy; diabetes mellitus; autoimmune diseases; malignant tumors; cardiovascular diseases (e.g., arrhythmia, atrial fibrillation, myocardial infarction, heart failure); stroke or peripheral artery disease; inflammatory bowel disease; receipt of renal replacement therapy (hemodialysis, peritoneal dialysis, or kidney transplantation); use of antibiotics, probiotics, or prebiotics, or gastrointestinal surgery within the past month; and history of gastrointestinal surgery within the preceding 3 months. CKD was diagnosed in subjects with abnormal renal structure or function according to the KDIGO Clinical Practice Guidelines (5). For all CKD patients, complete 24-h urine samples were collected to measure total urine volume, and protein concentration in mixed urine was determined to calculate 24-h total protein excretion. Massive proteinuria was defined as 24-h urinary protein excretion >3.5 g/24 h (5). CKD patients were further stratified into two groups based on 24-h urinary protein excretion: PROU-L group, 300 mg < 24 h urinary protein < 3,500 mg (*n* = 49); PROU-M group, 24-h urinary protein > 3,500 mg (*n* = 45).

### Sample collection

Fresh fecal samples were collected from all participants using sterile cryopreservation tubes. Samples were transported to the laboratory on dry ice and stored at −80°C for long-term preservation.

Demographic characteristics, including age, sex, and body mass index, were recorded. Clinical indicators were collected, comprising average office blood pressure, 24-h urinary protein quantification, uric acid, blood urea nitrogen, creatinine, estimated glomerular filtration rate, total cholesterol, triglycerides, low-density lipoprotein, high-density lipoprotein, hemoglobin, white blood cell count, platelet count, and albumin (Table 1).

**TABLE 1:** General clinical characteristics of study participants^*a*^

Characteristics	HC	PROU-L	PROU-M	P_HC vs PROU-L_	P_HC vs PROU-M_
Number	54	49	45	NA^*c*^	NA
Age, years	41.00 (32.75–50.00)	44.00 (33.00–53.00)	48.00 (34.00–59.00)	0.739	0.104
Male (%)	28	28	26	0.693	1.000
Systolic pressure, mmHg	116.50 (110.00–125.00)	133.67 (122.00–138.67)	134.67 (126.67–144.33)	<0.001	<0.001
Diastolic pressure, mmHg	70.00 (65.75–80.00)	80.33 (75.00–85.67)	79.67 (74.17–88.67)	<0.001	<0.001
BMI, kg/m^2^	23.08 (21.25–25.05)	24.63 (23.00–26.33)	24.49 (22.40–26.27)	0.010	0.035
TC, mmol/L	4.75 (4.21–5.31)	5.45 (4.71–6.04)	7.85 (6.54–9.73)	0.002	<0.001
HDL-C, mmol/L	1.40 (1.24–1.58)	1.23 (1.07–1.47)	1.47 (1.12–1.72)	0.026	0.654
TG, mmol/L	3.05 (2.53–3.88)	3.68 (2.86–4.35)	5.33 (3.48–7.03)	0.020	<0.001
LDL-C, mmol/L	0.96 (0.65–1.38)	1.30 (0.93–2.00)	1.77 (1.29–3.55)	0.002	<0.001
Blood urea nitrogen, mmol/L	5.06 (4.46–5.90)	5.02 (4.26–6.59)	4.92 (4.31–6.49)	0.693	0.896
Creatinine, umol/L	59.60 (51.30–70.65)	74.10 (61.00–96.95)	64.30 (55.85–80.85)	0.002	0.046
eGFR, mL/min/1.73 m²	111.25 (102.60–118.63)	101.59 (79.02–112.39)	100.12 (92.10–112.00)	<0.001	0.003
Uric acid, mmol/L	312.50 (247.50–380.75)	394.00 (322.00–453.50)	352.00 (298.00–435.50)	0.001	0.011
Urine total protein, G/24 h	0.00 (0.00–0.00)	1,600.00 (1,013.50–2,213.00)	6,169.00 (4,634.50–8,803.00)	<0.001	<0.001
Red blood cell*10^12^/L^*b*^	5.83 (5.13–8.02)	6.57 (5.23–7.74)	6.27 (5.09–7.41)	0.453	0.795
Hemoglobin, G/L	141.00 (132.00–153.25)	135.00 (122.00–146.50)	134.00 (119.00–143.00)	0.043	0.005
Platelets*10^9^/L	244.50 (218.00–288.50)	234.00 (186.00–281.00)	239.00 (200.50–307.50)	0.161	0.914
ARB/ACE-I (*n*)	NA	24	23	NA	NA
CCB (*n*)	NA	11	13	NA	NA
B-Block (*n*)	NA	6	6	NA	NA
Diuretics (*n*)	NA	5	15	NA	NA
Statins (*n*)	NA	21	34	NA	NA

^*a*^ HC, healthy controls; PROU-L, proteinuria-light; PROU-M, proteinuria-massive; BMI, body mass index; TC, total cholesterol; HDL-C, high-density lipoprotein cholesterol; TG, high-density lipoprotein cholesterol; LDL-C, low-density lipoprotein cholesterol; eGFR, estimated glomerular filtration rate.

^*b*^ “*” indicates that the corresponding cell counts are presented in units of ×10ⁿ/L.

^*c*^ NA, not applicable.

### 16S rRNA sequencing

DNA was extracted from fecal samples, and quantitative analysis was performed using Nanodrop technology (22). DNA integrity was assessed by 1.2% agarose gel electrophoresis, while concentration and purity were determined using a NanoDrop 2000 spectrophotometer (Thermo Fisher Scientific), ensuring an A260/A280 ratio >1.8. The V3–V4 variable regions of the 16S rRNA gene were amplified by polymerase chain reaction (PCR) using primers 338F (5′-ACTCCTACGGGAGGCAGCA-3′) and 806R (5′-GGACTACHVGGGTWTCTAAT-3′). Amplified products were quantified using the Quant-iT PicoGreen dsDNA Assay Kit in conjunction with a BioTek FLx800 microplate reader. PCR conditions were as follows: initial denaturation at 98°C for 2 min; 25 cycles of denaturation at 98°C for 15 s, annealing at 55°C for 30 s, and extension at 72°C for 30 s; followed by a final extension at 72°C for 5 min and a hold at 10°C. Library construction was performed using the Illumina TruSeq Nano DNA LT Library Prep Kit. Libraries were purified by 2% agarose gel electrophoresis and assessed for quality using an Agilent 2100 Bioanalyzer with the Agilent High Sensitivity DNA Kit. Libraries were subsequently quantified by the Qubit method and subjected to paired-end 250-bp (PE250) sequencing on the Illumina NovaSeq 6000 platform.

### Taxonomic annotation

Raw sequencing data were filtered using QIIME2 software (version 2019.4). Primer sequences were removed using the qiime cutadapt trim-paired plugin, with 338F (5ʹ-ACTCCTACGGGAGGCAGCA-3ʹ) as the forward primer and 806R (5ʹ-GGACTACHVGGGTWTCTAAT-3ʹ) as the reverse primer, and reads lacking either primer were discarded. Quality control, denoising, paired-end merging, and chimera removal were performed using the qiime dada2 denoise-paired plugin with default parameters. Quality filtering employed the expected error model implemented in DADA2, and chimeric sequences were removed using the consensus method. After denoising, amplicon sequence variants (ASVs) were generated and merged across sequencing libraries. Singleton ASVs were removed to minimize potential sequencing noise. Features annotated as mitochondria, chloroplast, or unassigned at the kingdom level were filtered prior to downstream analyses. Taxonomic annotation was performed using the Greengenes database (version 13.8; http://greengenes.lbl.gov) with the classify-sklearn method (https://github.com/QIIME2/q2-feature-classifier) (23). Taxonomic levels of annotation included phylum, class, order, family, and genus. Additionally, annotation was repeated in the SILVA database (release 138.1) to evaluate robustness to taxonomy reference choice (Tables S1 to S3).

### Microbial diversity analysis

Alpha diversity was assessed using the Chao1, Shannon, Simpson, Pielou’s evenness, and Good’s coverage indices, each calculated in QIIME2 (version 2019.4) (24). The Chao1 index, a non-parametric estimator of microbial richness, was computed using the qiime diversity alpha command with the chao1 metric. Shannon and Simpson indices were calculated using the same command with the respective Shannon and Simpson metrics. Pielou’s evenness was derived by dividing the Shannon index H' by the natural logarithm of the total number of ASVs. Good’s coverage index was calculated on the non-rarefied ASVs table using the goods_coverage metric to evaluate the proportion of sequences not represented by singletons. Beta diversity analysis was conducted to assess the dissimilarity between samples (25). A Bray-Curtis distance matrix was generated in QIIME2, calculating the distance between two samples as the sum of the absolute differences in species abundances divided by the total abundance. Beta diversity was further assessed using Aitchison distance derived from centered log-ratio (CLR)-transformed abundances to account for compositionality. Inter-group differences were visualized by principal component analysis (PCA) and principal coordinate analysis (PCoA) based on the distance matrices. Analysis of similarities (ANOSIM) and permutational multivariate analysis of variance (PERMANOVA) tests were applied to evaluate the statistical significance of these differences. Visualizations and statistical analyses were performed using the vegan and ggplot2 packages in R software. To compare sample distributions along the first principal coordinate axis, coordinate values for each sample were extracted. Inter-group differences in these axis scores were assessed using the Kruskal–Wallis test for multiple group comparisons, followed by pairwise Wilcoxon rank-sum tests where applicable, with significance defined as *P* < 0.05 (26). K-means clustering was performed using the OmicStudio tools (https://www.omicstudio.cn/tool/102) (27).

### Functional annotation

PICRUSt2 was employed to predict the potential functions of the gut microbial community (28). A phylogenetic tree was constructed based on the Greengenes database, and the genes and functions of the gut microbiota were inferred using the Kyoto Encyclopedia of Genes and Genomes (KEGG) to annotate metabolic pathways and enzyme activities. The 16S rRNA gene sequences of known microbial genomes were aligned and compared with the Greengenes database to construct a phylogenetic tree and infer gene functions of common ancestors. Using the Castor algorithm, copy numbers of gene families in the characteristic sequences were inferred by identifying the closest species based on gene family copy numbers in reference sequences within the phylogenetic tree (sequences with NSTI > 2 were excluded). Gene family copy numbers were then calculated by combining the abundance of characteristic sequences in samples, followed by hierarchical processing and integration of species information to enable functional-species association analyses. Gene families were mapped to databases such as KEGG, and MinPath was applied by default to infer metabolic pathways, thereby obtaining pathway abundance and associated information for each sample. By linking bacterial community composition with reference databases, functional prediction of gut bacterial genes was achieved.

### Statistical analysis

Inter-group comparisons were performed using the Kruskal–Wallis test for multiple groups and the Wilcoxon rank-sum test for pairwise comparisons, with significance defined as *P* < 0.05. Mfuzz analysis and random forest modeling were conducted using OmicStudio tools (https://www.omicstudio.cn/tool/121) in R software (version 4.1.3) with the Mfuzz package (version 2.54.0), randomForest package (version 4.7-1.1), OmicStudioClassic package (1.41.0), and OmicStudioKits package (1.41.0). Differential species and functional analysis were performed using linear discriminant analysis effect size (LEfSe) analysis (OmicStudio tools, https://www.omicstudio.cn/tool), with screening criteria set as linear discriminant analysis (LDA) score > 2 and *P* < 0.05. To account for compositional constraints, genus-level differential abundance was additionally assessed using CLR transformation, followed by Kruskal-Wallis tests with FDR correction. Spearman rank correlation analysis (using the corrplot package in R) was conducted to evaluate correlations between species and between microbiota and clinical parameters. Ternary plots were generated using the OmicStudio tools (https://www.omicstudio.cn/tool/147). Correlation networks were constructed in R (version 3.6.3) using the igraph package (1.2.6). Chord diagrams were plotted in R (version 4.1.3) on the OmicStudio platform using the ggplot2 package (3.3.3) at https://www.omicstudio.cn. Heatmaps were generated using the pheatmap package in R. Abundance profiles were transformed into Z scores by subtracting the mean abundance and dividing by the standard deviation across all samples. Z scores were visualized using a blue–red scale, with negative values in blue representing abundance below the mean and positive values in red representing abundance above the mean.

## RESULTS

### Baseline characteristics of participants

The study cohort comprised 54 HC, 49 patients in the PROU-L group, and 45 patients in the PROU-M group. Baseline characteristics are presented in Table 1. Compared with the HC group, patients in the PROU-L and PROU-M groups exhibited significantly higher systolic blood pressure (SBP), diastolic blood pressure (DBP), body mass index (BMI), total cholesterol (TC), triglycerides (TG), low-density lipoprotein (LDL), creatinine, uric acid, and urine total protein, alongside significantly lower eGFR, fasting blood glucose, and hemoglobin (all *P* < 0.05). These differences correspond with the progressive increase in proteinuria observed in CKD patients, which is associated with declining renal function, hypoproteinemia, and reduced colloid osmotic pressure, resulting in edema and subsequent sodium-water retention that contributes to elevated blood pressure. Additionally, increased proteinuria induces metabolic disturbances accompanied by deteriorating nutritional status, which may underlie the gradual decline in hemoglobin.

### Gut microbiota composition in patients with different stages of proteinuria

**Fig 1:**
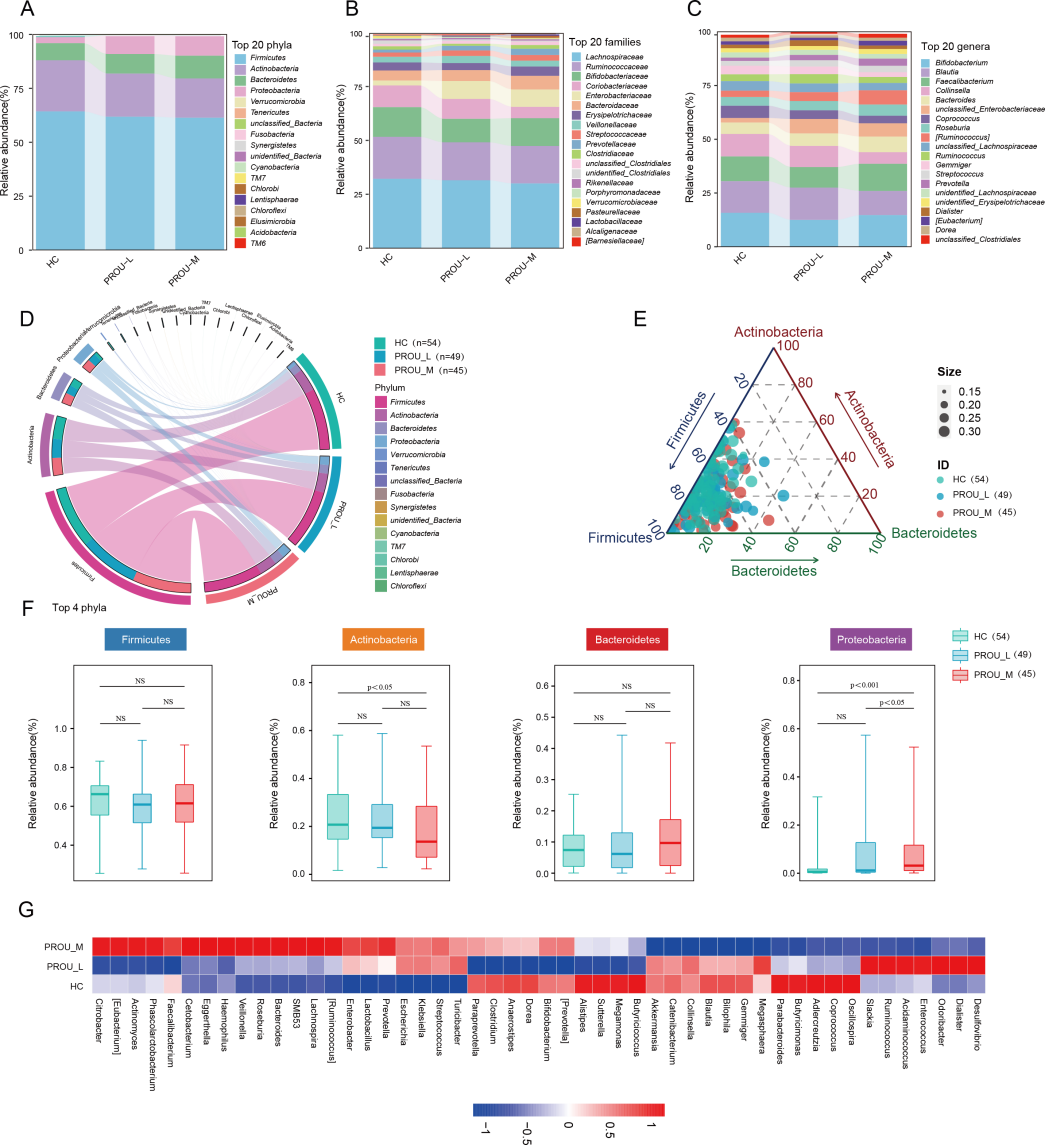
Global characteristics of gut microbiota in patients with different stages of proteinuria. (**A–C**) The gut microbiota composition of the HC, PROU-L, and PROU-M groups. The top 20 phyla, families, and genera in terms of abundance are shown with their relative abundance proportions. (**D**) Chord diagram showing the proportions of various microbial phyla among the HC, PROU-L, and PROU-M groups. (**E**) The relative abundances of the top three dominant phyla (p__Firmicutes, p__Actinobacteria, p__Bacteroidetes) in the three groups. (**F**) Comparison of abundance differences of the top four phyla (p__Firmicutes, p__Actinobacteria, p__Bacteroidetes, p__Proteobacteria) among the three groups, showing differences between each pair of groups. Wilcoxon Rank-sum test (*P < 0.05, **P < 0.01, ***P < 0.001). Box plots represent the interquartile range; the inner line represents the median. (**G**) The heatmap depicts the relative abundance and distribution of the most abundant genera across groups. The abundance values were transformed into Z-scores by subtracting the mean and dividing by the standard deviation across all samples. Rows with a Z-score below the mean (negative values) are shown in blue, while those above the mean (positive values) are shown in red.

Distinct gut microbiota distributions were observed among the HC, PROU-L, and PROU-M groups, with compositional differences evident at the phylum, family, and genus levels. At the phylum level, the relative abundances of p__Fusobacteria (HC: 0.03%; PROU-L: 0.05%; PROU-M: 0.06%), p__Proteobacteria (HC: 2.79%; PROU-L: 8.63%; PROU-M: 9.35%), and p__Bacteroidetes (HC: 7.93%; PROU-L: 9.05%; PROU-M: 10.57%) increased progressively from the HC group to the PROU-L group and further to the PROU-M group (Fig. 1A). In contrast, the abundances of p__Firmicutes (HC: 64.32%; PROU-L: 61.88%; PROU-M: 61.40%) and p__Actinobacteria (HC: 23.71%; PROU-L: 20.00%; PROU-M: 18.19%) showed a progressive decreasing trend. These phylum-level changes, however, did not reach statistical significance in the overall comparison across the three groups (*P* > 0.05, Kruskal-Wallis test with FDR correction) in the overall comparison across the three groups. At the family level, the abundances of f__Streptococcaceae (HC: 1.93%; PROU-L: 2.53%; PROU-M: 2.60%) and f__Prevotellaceae (HC: 1.32%; PROU-L: 2.15%; PROU-M: 2.88%) increased gradually with disease severity, whereas the abundances of f__Lachnospiraceae (HC: 31.89%; PROU-L: 31.19%; PROU-M: 29.74%), f__Ruminococcaceae (HC: 19.27%; PROU-L: 17.44%; PROU-M: 17.19%), and f__Coriobacteriaceae (HC: 9.95%; PROU-L: 9.11%; PROU-M: 5.22%) decreased sequentially (Fig. 1B). At the genus level, the abundances of g__Streptococcus (HC: 1.92%; PROU-L: 2.52%; PROU-M: 2.60%), g__Ruminococcus (HC: 2.55%; PROU-L: 3.57%; PROU-M: 5.72%), g__Bacteroides (HC: 4.60%; PROU-L: 5.00%; PROU-M: 6.26%), and g__Prevotella (HC: 1.32%; PROU-L: 2.15%; PROU-M: 2.88%) increased progressively across groups. In contrast, the abundances of g__Collinsella (HC: 9.00%; PROU-L: 8.50%; PROU-M: 4.64%), g__Gemmiger (HC: 3.44%; PROU-L: 3.07%; PROU-M: 1.89%), g__unclassified_Lachnospiraceae (HC: 2.10%; PROU-L: 2.02%; PROU-M: 1.87%), and g__Dorea (HC: 1.47%; PROU-L: 1.28%; PROU-M: 1.23%) gradually decreased (Fig. 1C). Further analysis revealed that at the phylum level, the HC group was dominated by Firmicutes and Actinobacteria, whereas the proportion of potentially pathogenic bacteria, such as Proteobacteria and Fusobacteria, increased in the PROU-M group (Fig. 1D).

Ternary plot analysis indicated that the relative abundance of Bacteroidetes increased progressively from HC (7.94%) to PROU-L (9.05%) and PROU-M (10.58%), while Actinobacteria decreased from HC (23.71%) to PROU-L (20.00%) and PROU-M (18.19%). Firmicutes also showed a decreasing trend across the three groups (HC: 64.32%; PROU-L: 61.88%; PROU-M: 61.40%) (Fig. 1E). Significant differences were observed in the top four dominant phyla among groups. Compared with HC, Actinobacteria abundance was significantly reduced in PROU-M (*P* = 0.02). Conversely, Proteobacteria abundance increased progressively with proteinuria severity, showing statistically significant differences between HC and PROU-M (*P* < 0.001) and between PROU-L and PROU-M (*P* < 0.05) (Fig. 1F). These findings indicate that worsening proteinuria is associated with a progressive increase in Proteobacteria and a decrease in Actinobacteria. At the genus level, beneficial bacteria, such as Akkermansia, Butyricicoccus, and Coprococcus, were reduced in the PROU-L and PROU-M groups, whereas opportunistic pathogens, including Streptococcus, Klebsiella, Turicibacter, and Escherichia, were increased (Fig. 1G).

### Microbial diversity and alterations in gut microbiota structure in patients with different stages of proteinuria

**Fig 2:**
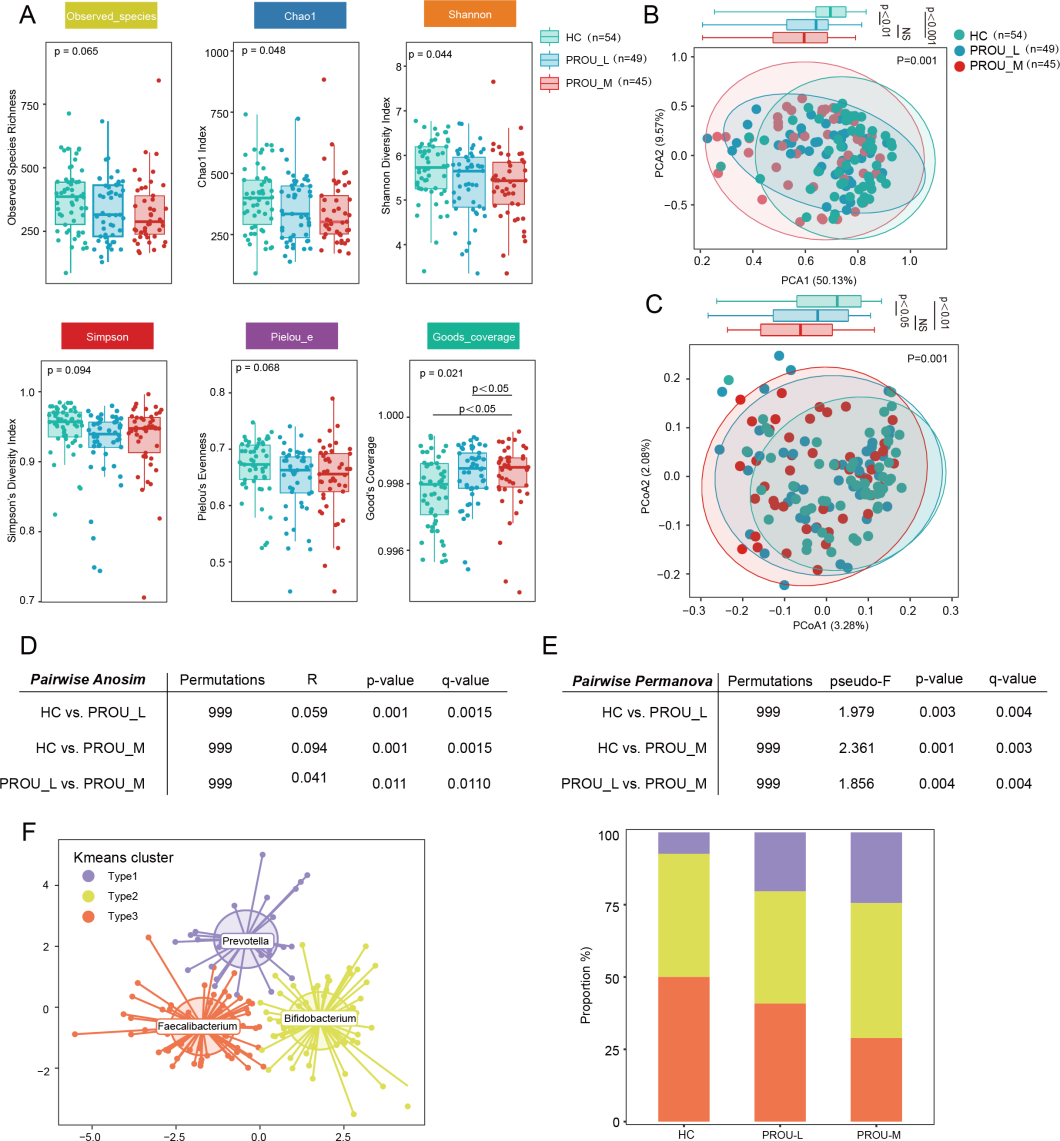
Microbial diversity and gut microbiota structure in patients with different stages of proteinuria. (**A**) Differences in alpha diversity of gut microbiota among the HC, PROU-L, and PROU-M groups, including observed species, Chao richness index, Shannon diversity index, Simpson index, Goods_coverage index, and Pielou evenness index. *P* values were calculated using the Kruskal-Wallis rank-sum test. Box plots represent the interquartile range; the inner line represents the median. (**B and C**) PCA and PCoA based on Bray-Curtis distance to analyze the heterogeneity of subjects among the HC, PROU-L, and PROU-M groups. Different colors in the scatter plots represent samples from different groups. The higher the similarity between samples, the closer they are distributed in the figure. *P* values were calculated using ANOSIM to assess the significant differences in the total variability explained by each principal coordinate. Box plots show the distribution of individual axes in PCA1 and PCOA2, representing the interquartile range; the inner line represents the median. (**D and E**) Comparison of beta diversity between groups, with significant differences calculated using ANOSIM and PERMANOVA, respectively. (**F**) Microbiota samples of all subjects were clustered into types using K-means clustering based on genus-level features. Calinski-Harabasz index was presented, and the percentage distribution of HC, PROU-L, and PROU-M groups in Type 1, Type 2, and Type 3 was calculated. In Type 1, HC: 7.41%, PROU-L: 20.41%, PROU-M: 24.44%; in Type 2, HC: 42.59%, PROU-L: 38.78%, PROU-M: 46.67%; in Type 3, HC: 50.00%, PROU-L: 40.82%, PROU-M: 28.89%.

In order to compare changes in microbial community diversity among the HC, PROU-L, and PROU-M groups, we next evaluated alpha and beta diversity indices of gut microbiota at the genus level, including the number of species, Chao1 richness index, Shannon diversity index, Simpson index, Goods_coverage index, and Pielou evenness index. Compared with the HC group, alpha diversity in the PROU-L and PROU-M groups was reduced. Statistically significant differences were observed among the three groups for the Chao1 index (*P* = 0.048), Shannon index (*P* = 0.044), and Good’s coverage index (*P* = 0.021). Although the Simpson index, Pielou’s evenness, and observed species did not reach statistical significance, a decreasing trend was apparent across groups (Fig. 2A).

Beta diversity of gut microbial communities was assessed using PCA and PCoA. Both analyses revealed significant differences among groups (PPCA = 0.001; PPCoA = 0.001). Distribution along the first axis showed significant separation between HC and PROU-L, and between HC and PROU-M. Specifically, for HC vs PROU-L, PPCA1 < 0.01 and PPCoA1 < 0.05; for HC vs PROU-M, PPCA1 < 0.001 and PPCoA1 < 0.01 (Fig. 2B and C), with differences intensifying progressively with proteinuria severity. ANOSIM and PERMANOVA confirmed significant inter-group differences (Fig. 2D and E), with the HC group differing significantly from both PROU-L and PROU-M, and the q value between PROU-L and PROU-M being <0.05. To address the compositional nature of microbiome data, β-diversity was further assessed using Aitchison distance derived from CLR-transformed abundances, yielding consistent results (PERMANOVA, pseudo-F = 2.472, *P* = 0.001), with homogeneity of dispersion confirmed (PERMDISP, F = 1.914, *P* = 0.164).

K-means clustering of 148 samples identified three enterotypes (Fig. 2F), dominated by Prevotella (Type 1), Bifidobacterium (Type 2), and Faecalibacterium (Type 3) at the genus level. The results demonstrated that Type 1 increased and Type 3 decreased sequentially from the HC to the PROU-L and PROU-M groups. For Type 1, the PROU-M group exhibited the highest proportion (HC: 7.41%; PROU-L: 20.41%; PROU-M: 24.44%). In contrast, for Type 3, the highest proportion was observed in the HC group (HC: 50.00%; PROU-L: 40.82%; PROU-M: 28.89%).

### Core gut microbial markers in patients with different stages of proteinuria

**Fig 3:**
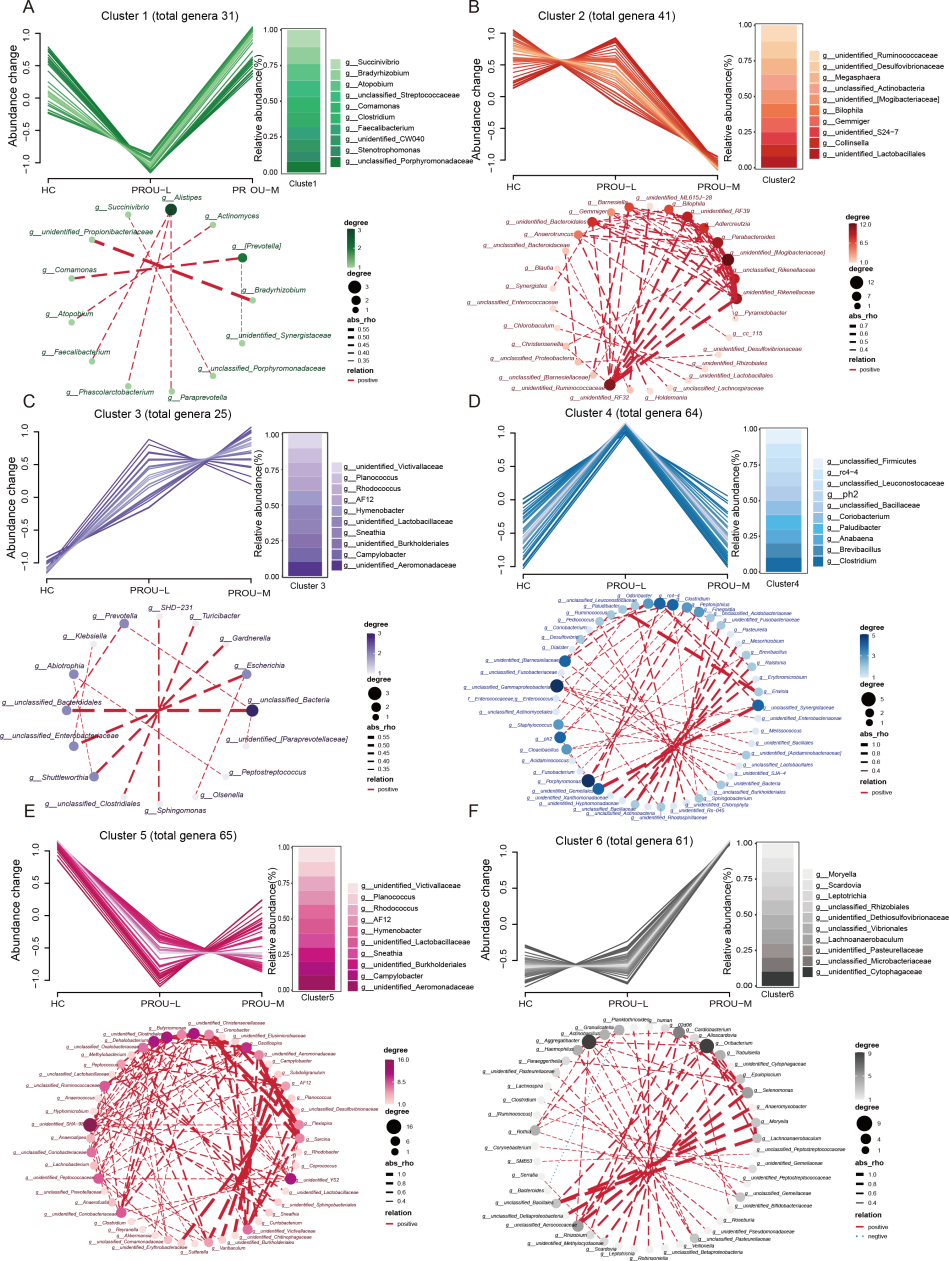
Abundance distribution of gut microbial genera in patients with different stages of proteinuria. (**A**) Line graphs of genera generated by Mfuzz analysis show that six clusters exhibit different enrichment patterns from the HC group to the PROU-L group and then to the PROU-M group. Cluster 1 contains 31 genera, with their average abundance changes among the HC, PROU-L, and PROU-M groups (X-axis); the Y-axis represents normalized abundance. The bar chart shows the composition of the top 10 most abundant genera in cluster 1, and the co-occurrence network of genera within this cluster is analyzed: the thickness of the connecting lines is based on the Spearman correlation |r| value, and the color and size of nodes correspond to their connectivity. (**B–F**) The total number of genera included in clusters 2–6 is marked, and the abundance changes in the line graphs represent normalized levels. (**B**) Cluster 2; (**C**) cluster 3; (**D**) cluster 4; (**E**) cluster 5; (**F**) cluster 6. Bar charts show the relative abundances of the most abundant (top 10) genera in each differential cluster. The interactions further display the network among genera in each cluster.

Mfuzz clustering analysis was performed to investigate gut microbiota composition based on genus-level abundance changes from HC to PROU-L and then to PROU-M (Fig. 3). Six differential clusters were identified according to genus abundance patterns. Cluster 2 contained 41 genera, dominated by unidentified_Ruminococcaceae, unidentified_Desulfovibrionaceae, and Megasphaera, which decreased progressively across groups (Fig. 3B). Cluster 3 included 25 genera, mainly Enterobacter, [Clostridium], Gardnerella, and unclassified_Bacteroidales, which increased progressively with proteinuria severity (Fig. 3C). Cluster 5 comprised 65 genera, such as unidentified_Victivallaceae, Planococcus, Rhodococcus, and AF12, which were more abundant in HC and significantly decreased in PROU-L and PROU-M (Fig. 3E). Cluster 6 contained 61 genera, including Moryella, Scardovia, Leptotrichia, and unclassified_Rhizobiales, which were most abundant in PROU-M and significantly reduced in HC and PROU-L (Fig. 3F).

Cluster 1 included 31 genera, such as Succinivibrio, Bradyrhizobium, Atopobium, and unclassified_Streptococcaceae (Fig. 3A), whereas cluster 4 comprised 64 genera (including unclassified_Firmicutes, rc4-4, unclassified_Leuconostocaceae, and ph2) that exhibited increased abundance in PROU-L and reduced abundance in HC and PROU-M (Fig. 3D). Clusters 2 and 3 displayed progressive depletion or enrichment from HC to PROU-L to PROU-M, while clusters 5 and 6 highlighted genera specifically altered in PROU-M, suggesting potential microbial markers. Complex interactions were observed among genera in clusters 1, 4, 5, and 6, showing strong associations (Fig. 3A, D through F), whereas genera in clusters 1 and 3 exhibited weaker correlations.

### Analysis of differential gut microbiota in patients with different stages of proteinuria

**Fig 4:**
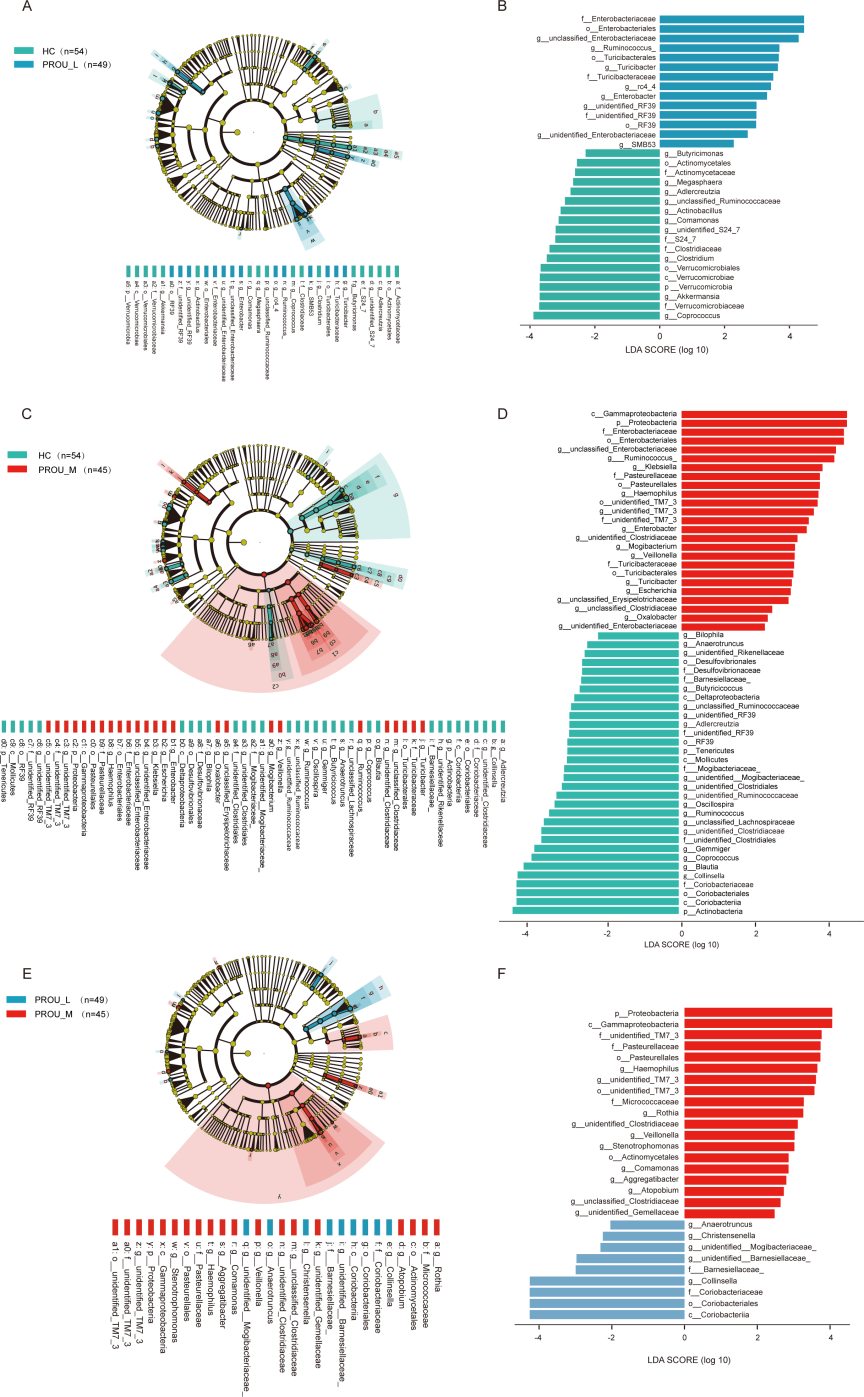
Analysis of differential gut microbiota among the HC, PROU-L, and PROU-M groups. (**A and B**) LEfSe analysis showing the composition of differential taxa between the HC group and the PROU-L group. The phylogenetic tree and bar chart of LDA scores display the taxa significantly enriched in different groups. The statistical significance criteria for different bacterial taxa are LDA score (log10) > 2 and *P* value < 0.05. (**C and D**) LEfSe analysis showing the taxa with significant differences in bacterial abundance between the HC group and the PROU-M group. (**E and F**) LEfSe analysis showing the taxa with significant differences in bacterial abundance between the PROU-L group and the PROU-M group.

To identify key microbial markers in patients with proteinuria compared with healthy controls, gut microbiota differences among PROU-L, PROU-M, and HC subjects were analyzed using LEfSe (Fig. 4; Table S4). Between the HC and PROU-L groups, 32 significantly different species were identified. Of these, 14 were enriched in the PROU-L group, including f__Enterobacteriaceae (which produces endotoxin [LPS], exacerbates renal oxidative stress, and contributes to urinary tract infections); g__Ruminococcus and g__Turicibacter (which produce pro-inflammatory metabolites); and g__Enterobacter (which produces the nephrotoxic metabolite phenylacetic acid). The remaining 18 were enriched in the HC group, including probiotics, such as g__Akkermansia (which produces acetic acid and propionic acid, inhibiting renal interstitial fibrosis), and g__Coprococcus and g__Butyricicoccus (which produce butyrate) (Fig. 4A and B).

Between the HC and PROU-M groups, 57 significantly different species were identified. Among these, 25 were enriched in the PROU-M group, including f__Enterobacteriaceae, g__Haemophilus (which induces immune-mediated renal injury via deposition of antigen-antibody complexes, leading to hematuria, proteinuria, and reduced renal function), g__Ruminococcus, and g__Turicibacter. The remaining 32 species were enriched in the HC group, including g__Adlercreutzia (associated with cardiovascular health and anti-inflammatory effects), g__Blautia (which produces SCFAs, such as acetic acid and propionic acid), and g__Coprococcus and g__Butyricicoccus (Fig. 4C and D). Comparison between the PROU-L and PROU-M groups identified eight significantly differential genera, primarily inflammation-associated taxa, such as g__Stenotrophomonas, f__Pasteurellaceae, and g__Haemophilus (Fig. 4E and F). Genus-level differential abundance was also reassessed using a CLR-based approach to account for compositionality (Table S5). Notably, key taxa identified by LEfSe, including g__Haemophilus, g__Ruminococcus, g__Turicibacter, and g__Enterobacter, remained significant after FDR correction, supporting the robustness of these findings.

Based on LEfSe results, analysis of common differential bacteria between HC vs PROU-L and PROU-L vs PROU-M revealed 18 genera with significant changes (Fig. S1A). Correlation analysis between these 18 genera and clinical characteristics showed significant associations with laboratory indicators (Fig. S1B). For example, the abundance of c__Coriobacteriia decreased progressively from HC to PROU-L and PROU-M and was negatively correlated with SBP, LDL, and eGFR, while positively correlated with fasting blood glucose. In contrast, p__Proteobacteria abundance increased progressively across the three groups and was positively correlated with TG, LDL, blood urea nitrogen, serum creatinine, uric acid, and DBP, while negatively correlated with eGFR. These findings indicate that gut microbiota dysbiosis may contribute to the pathogenesis and progression of renal dysfunction, hypertension, hyperlipidemia, and other pathophysiological conditions in patients with proteinuria.

### Functional analysis of gut microbiota in patients with different stages of proteinuria

**Fig 5:**
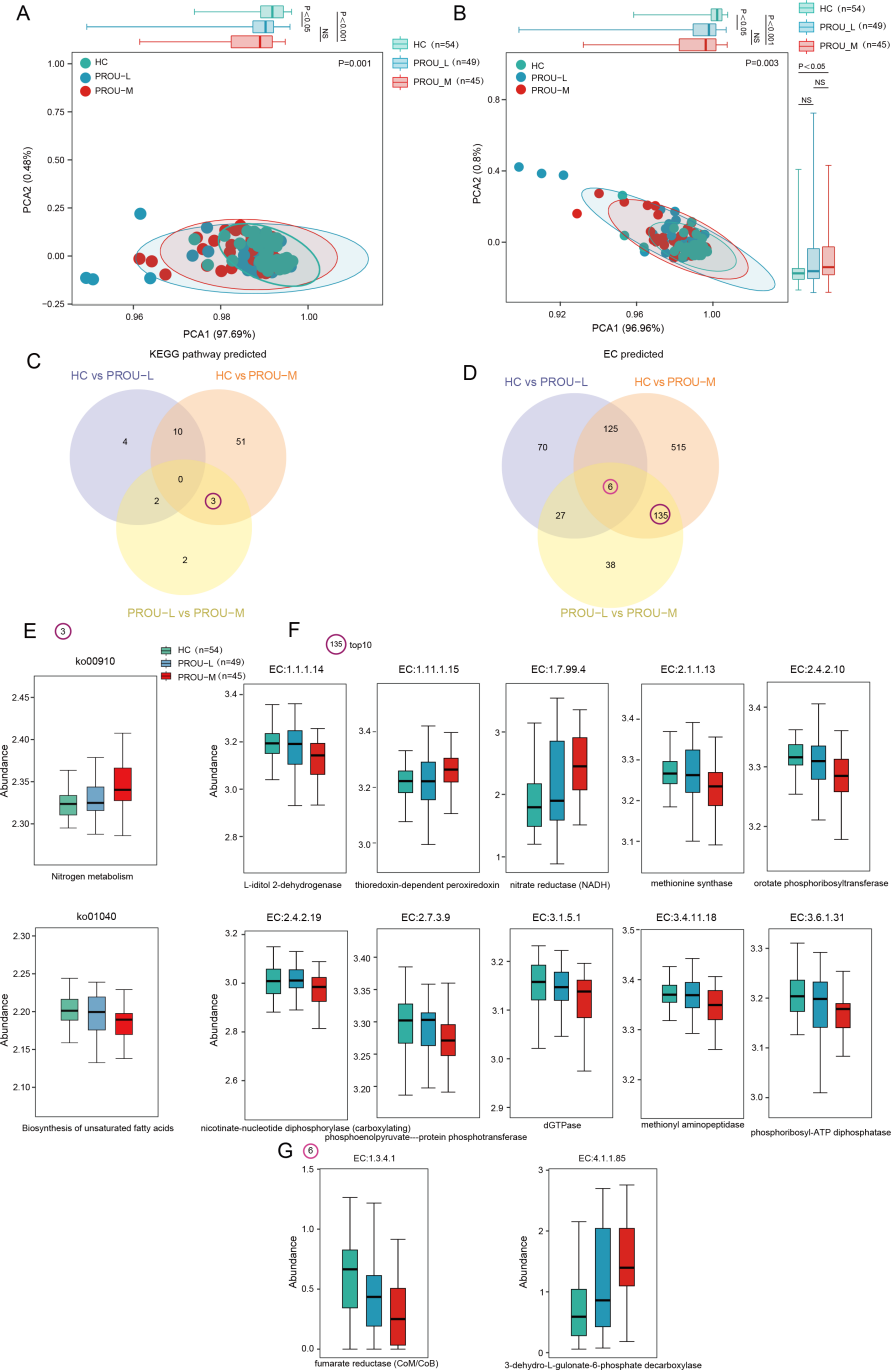
Functional changes of gut microbiota in patients with different proteinuria stages based on KEGG analysis. (**A and B**) PCA scatter plots generated from PICRUSt2-predicted KEGG pathway (**A**) and EC (**B**) features. *P* values from the ANOSIM test were used to evaluate the significance of heterogeneity among the HC, PROU-L, and PROU-M groups. Box plots show the distribution differences of each group on PCA principal coordinates 1 and 2, and the Wilcoxon rank-sum test was used to compare inter-group differences. (**C and D**) Venn diagrams showing the intersection of differential microbial KEGG pathways (**C**) and ECs (**D**) in three group comparisons: HC group vs PROU-L group; HC group vs PROU-M group; PROU-L group vs PROU-M group. (**E, F, and G**) Box plots showing the abundance distribution of shared differential KEGG pathways (**E**) and ECs (**F and G**) among different comparison groups in the HC, PROU-L, and PROU-M groups. Box plots represent the interquartile range, with the inner horizontal line as the median. (**E**) Shared changed KEGG pathways between the HC group vs PROU-M group and PROU-L group vs PROU-M group. (**F**) Top 10 of 135 shared changed ECs between the HC group vs PROU-M group and PROU-L group vs PROU-M group. (**G**) Shared ECs in pairwise comparisons among the three groups.

Based on the KEGG database, the functional potential of the gut microbiota in each group was evaluated, annotating 174 KEGG metabolic pathways and 2,110 enzyme complexes (ECs) (Tables S6 to S11). PCA and ANOSIM based on KEGG pathways and ECs revealed significant differences among the HC, PROU-L, and PROU-M groups (PPCA for KEGG = 0.001; PPCA for ECs = 0.003). Further analysis of inter-group differences showed that both KEGG pathways and ECs exhibited significant differences in sample distribution along PCA1 between HC vs PROU-L and HC vs PROU-M (KEGG: PHC vs PROU-L < 0.05; PHC vs PROU-M < 0.001; ECs: PHC vs PROU-L < 0.05; PHC vs PROU-M < 0.001), with differences progressively intensifying with increasing proteinuria severity (Fig. 5A and B). Using the Wilcoxon rank-sum test, 72 KEGG pathways and 916 ECs were found to differ significantly among groups (Fig. 5C and D). Between HC and PROU-M, 3 KEGG pathways and 135 ECs showed marked changes compared with those between PROU-L and PROU-M (Fig. 5C and D). For example, microbial functions related to nitrogen metabolism (ko00910) were specifically increased in the PROU-M group, whereas pathways associated with the biosynthesis of unsaturated fatty acids (ko01040) were decreased (Fig. 5E). Six ECs (EC:1.3.4.1, EC:1.7.2.5, EC:2.7.1.166, EC:4.1.1.85, EC:6.5.1.6, EC:6.5.1.7) exhibited synchronous changes across the three groups. For instance, EC:1.3.4.1 (fumarate reductase [CoM/CoB]) progressively decreased from the HC group to PROU-L and further to PROU-M, whereas EC:4.1.1.85 (3-dehydro-L-gulonate-6-phosphate decarboxylase) progressively increased (Fig. 5D through G). Certain ECs (e.g., EC:1.1.1.14, EC:2.4.2.19, EC:3.1.5.1, EC:2.4.2.10) were specifically reduced in the PROU-M group, whereas others (e.g., EC:1.11.1.15, EC:1.7.99.4, EC:2.1.1.13) were specifically increased in PROU-M (Fig. 5F).

**Fig 6:**
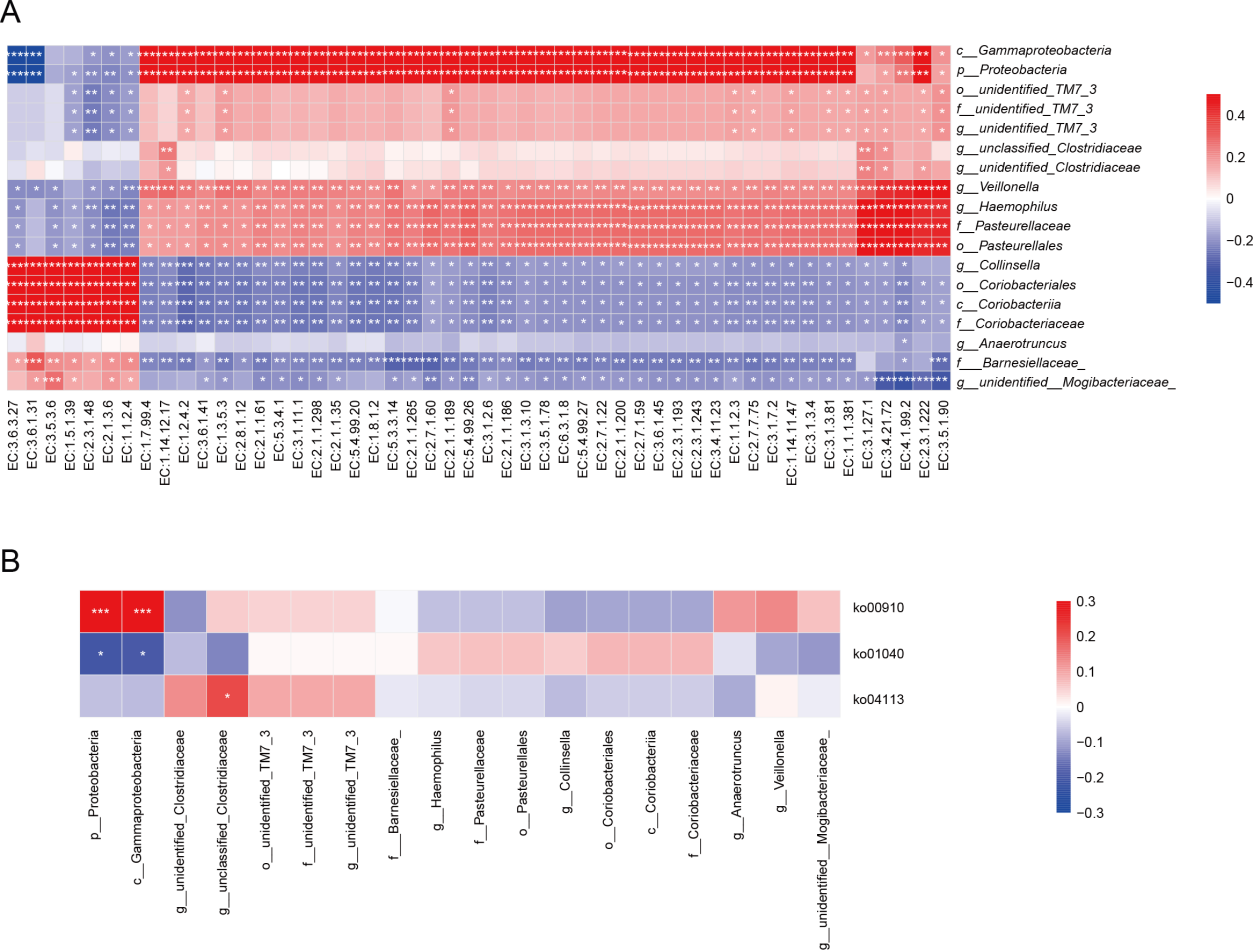
Correlation analysis between significantly differential gut genera and ECs, as well as KEGG pathways in patients with CKD and proteinuria. (**A**) Significantly different genera between the HC group vs PROU-M group and PROU-L group vs PROU-M group are listed, and Spearman correlation analysis was used to explore the potential associations between these key genera and core ECs. (**B**) Significantly different genera between the HC group vs PROU-M group and PROU-L group vs PROU-M group are listed, and Spearman correlation analysis was used to explore the potential associations between these key genera and core KEGG pathways. In the heatmap, red indicates a positive correlation, blue indicates a negative correlation, and the color intensity corresponds to the magnitude of the correlation coefficient |r|; statistical significance is marked as *P < 0.05 and **P < 0.01.

Spearman correlation analysis was performed to examine whether shifts in key genera, such as the enrichment of Proteobacteria and Haemophilus in higher proteinuria groups or the reduction of beneficial genera like Akkermansia, were associated with alterations in microbial functional pathways, including nitrogen metabolism (ko00910) and unsaturated fatty acid biosynthesis (ko01040). Significant correlations indicated that these differentially abundant bacteria may reflect the dysregulated metabolic state of the gut microbiota in CKD progression. For example, the positive correlation between Proteobacteria and nitrogen metabolism supports the hypothesis that pathogenic enrichment drives uremic toxin production, whereas the negative correlation with unsaturated fatty acid synthesis indicates a loss of anti-inflammatory metabolic capacity (Fig. 6A). Specifically, p__Proteobacteria and c__Gammaproteobacteria, enriched in the PROU-M group, were significantly negatively correlated with EC:3.6.1.31 (phosphoribosyl-ATP diphosphatase), while f___Barnesiellaceae, enriched in the HC group, showed a significant positive correlation with EC:3.6.1.31 (Fig. 6B). These key genera exhibited complex associations with KEGG pathways, particularly in the PROU-M group, suggesting that gut microbiota dysbiosis may contribute to disease progression by affecting metabolic processes.

### Development of the diagnostic model for classifying proteinuria levels in CKD

A random forest classifier was applied to genus-level abundance data, using proteinuria groups (HC, PROU-L, PROU-M) as the outcome variable (Tables S12 to S14). This model identified the most important microbial features driving classification, quantified their predictive performance via out-of-bag error or cross-validation accuracy, and highlighted key discriminatory genera, including Turicibacter, Coprococcus, Enterobacter, and Haemophilus. This approach provides an integrated perspective on microbiota-based risk stratification in CKD.

## DISCUSSION

Previous studies on the gut-kidney axis have primarily focused on advanced CKD or the uremic stage, largely neglecting the early stages of CKD, particularly the proteinuric phase. As a key diagnostic and prognostic indicator of CKD, the relationship between changes in proteinuria levels and gut microbiota dysbiosis remains incompletely understood. This study specifically targeted CKD patients with varying proteinuria grades, exploring the dynamic changes in gut microbiota composition and function associated with worsening proteinuria, thereby addressing a gap in research on early-stage CKD.

We observed that with increasing proteinuria, CKD patients exhibited progressive gut microbiota dysbiosis. The relative abundance of probiotic phyla, including Firmicutes and Actinobacteria, declined, whereas pathogenic phyla such as Proteobacteria increased. SCFA-producing probiotics, including Akkermansia, Coprococcus, and Adlercreutzia, were progressively reduced, and pathogenic bacteria, such as Enterobacteriaceae, Haemophilus, and Ruminococcus, were enriched. Additionally, a pronounced enterotype shift was evident: the healthy group was dominated by a Faecalibacterium-dominated enterotype, whereas the group with massive proteinuria shifted toward a potentially pathogenic Prevotella-dominated enterotype. These stage-specific microbial features may serve as potential biomarkers for proteinuria grading and CKD progression.

Using 16S rRNA sequencing, we characterized gut microbiota dysbiosis in CKD patients across different proteinuria stages and identified dynamic alterations in microbiota composition. At the phylum level, probiotic-associated taxa (Firmicutes and Actinobacteria) decreased, while pathogenic taxa (Proteobacteria and Fusobacteria) increased. At the genus level, SCFA-producing probiotics (e.g., Akkermansia, Coprococcus) were depleted, whereas inflammation-promoting and nephrotoxicity-associated bacteria (e.g., Enterobacteriaceae, Haemophilus) were enriched. These genera may serve as potential indicators of proteinuria progression. Dysbiosis of the gut microbiota may contribute to CKD progression through alterations in metabolic pathways, including enhanced nitrogen metabolism, which promotes the production of uremic toxins, and reduced unsaturated fatty acid synthesis, which diminishes anti-inflammatory capacity.

The association between gut microbiota dysbiosis and CKD pathogenesis is well established. Declining kidney function leads to the accumulation of uremic toxins and dietary restrictions (e.g., low-fiber intake), driving the overgrowth of urease-producing bacteria, such as Enterobacteriaceae, while depleting beneficial symbionts with anti-inflammatory properties, including Bifidobacterium and Lactobacillus (29–31). This dysbiosis exacerbates intestinal barrier disruption, promotes the translocation of endotoxins (e.g., LPS) and microbial metabolites (e.g., indoxyl sulfate, TMAO) into the circulation, and amplifies systemic inflammation, oxidative stress, and nephrotoxicity, thereby accelerating CKD progression (12, 17, 32). However, existing research has largely concentrated on ESRD and dialysis populations, consistently reporting a marked reduction in alpha diversity, a >50% decline in the abundance of Bifidobacterium and Lactobacillus, and an expansion of conditional pathogens. These alterations closely correlate with uremic toxin accumulation and diminished SCFA synthesis. In contrast, studies investigating early-stage CKD, particularly the proteinuric phases characteristic of conditions such as IgA nephropathy and diabetic nephropathy, remain limited. Available evidence suggests that reductions in Lactobacillus and Bifidobacterium, along with enrichment of Proteobacteria, may already occur during these early stages; however, the precise dysbiotic signatures, dynamic evolutionary patterns, and mechanistic links with proteinuria grading remain to be fully elucidated (32, 33).

Coprococcus, Akkermansia, and Adlercreutzia, as core intestinal flora or common symbiotic bacteria, exhibited a progressive decline in abundance with worsening proteinuria in this study, and their alterations were closely associated with disease progression. Coprococcus produces SCFAs, particularly acetic acid and butyric acid, through fermentation of dietary fiber. A reduction in its abundance is linked to various systemic diseases (34, 35). In CKD patients, including those with end-stage renal disease in previous studies and in the present cohort, Coprococcus abundance is markedly decreased. Butyric acid exerts potent anti-inflammatory effects by inhibiting histone deacetylase (HDAC) and activating G-protein coupled receptors. The depletion of butyrate-producing bacteria reduces intestinal butyrate levels, compromising the intestinal barrier, facilitating the translocation of gut-derived toxins into the circulation, and exacerbating renal inflammation and fibrosis (34–36). Animal studies have demonstrated that supplementation with butyrate-producing bacteria or a high-fiber diet can restore microbial balance and mitigate renal injury, suggesting potential therapeutic value (37). Akkermansia contributes to intestinal barrier integrity, reduces endotoxemia (e.g., decreasing LPS), regulates SCFA production and immune responses (38–40), and improves glucose metabolism and immunity through the G-protein coupled Receptor43 (GPR43) receptor (41).

In CKD, LPS translocation due to impaired barrier function activates the renal Toll-Like Receptor 4 (TLR4)/NF-κB pathway, promoting proteinuria and glomerulosclerosis (42). In this study, the marked reduction in Akkermansia abundance in the proteinuria groups (PROU-L/M) may exacerbate mucosal injury, systemic inflammation, and endotoxemia, thereby contributing to CKD progression. Akkermansia may represent a potential target for interventions aimed at restoring intestinal barrier function, reducing LPS, and modulating TMAO levels. Adlercreutzia possesses anti-inflammatory properties, and its metabolites (e.g., equol, SCFAs, palmitoyl serine) exert multiple regulatory effects on host health (43). Reduced Adlercreutzia abundance is associated with progression of non-alcoholic fatty liver disease and cirrhosis and is inversely correlated with vascular calcification in hemodialysis patients, suggesting that its metabolites may protect against inflammation (44, 45). In diabetic nephropathy models, Adlercreutzia can delay renal injury by increasing methylsuccinic acid levels (46). In this study, the significant decline in Adlercreutzia abundance in the proteinuria groups (PROU-L/M) may exacerbate microbial dysbiosis. A reduction in its metabolite acetic acid may activate the renin-angiotensin system, disrupt intestinal-renal axis homeostasis, and promote proteinuria and renal injury. Collectively, the concurrent decline of Coprococcus, Akkermansia, and Adlercreutzia amplifies CKD-related intestinal dysbiosis, characterized by impaired SCFA synthesis, compromised barrier function, and systemic inflammation. These changes may serve as biomarkers for proteinuria grading and provide a rationale for microecological interventions targeting the intestinal-renal axis, such as probiotic or dietary fiber supplementation.

In this study, the abundance of pathogenic bacteria, including Enterobacteriaceae, Haemophilus, and Ruminococcus, increased significantly with worsening proteinuria. Although Enterobacteriaceae constitute less than 1% of the microbiota in healthy individuals (47, 48), it is markedly enriched in CKD patients. This enrichment elevates uremic toxin levels (e.g., indole, p-cresol, oxalic acid) (36), consistent with our findings. Translocated LPS triggers systemic inflammatory responses, increases the risk of endotoxemia, and accelerates CKD progression (36, 49). As a TMAO precursor, Enterobacteriaceae induce vascular inflammation, renal fibrosis, and atherosclerosis via activation of the NF-κB/MAPK (Mitogen-Activated Protein Kinase) pathway (49). It also secretes promoters of calcium oxalate crystallization (iron/chloride), facilitating kidney stone formation (50). In diabetic nephropathy, Enterobacteriaceae proliferate abnormally through metabolic reprogramming, synergizing with these mechanisms to exacerbate disease progression (51). Its enrichment in the proteinuria groups (PROU-L/M) may drive renal function decline via toxin-inflammation-metabolism interactions. Haemophilus contributes to disease progression through pro-inflammatory effects, antigen deposition, and immune-mediated mechanisms (52, 53).

As a potential risk factor for kidney stones, it promotes stone formation via gut-kidney axis-mediated inflammation (54, 55). In diabetic nephropathy, Haemophilus accelerates renal injury by modulating metabolites (e.g., increasing arachidonoyl carnitine) (46). The outer membrane antigen of Haemophilus forms immune complexes with IgA, which deposit in the glomeruli, directly inducing matrix expansion and inflammatory responses (56, 57). Haemophilus may therefore contribute to CKD progression through combined pro-inflammatory, metabolic, and immune-mediated pathways. Ruminococcus is highly expressed in patients with inflammatory bowel disease and exacerbates inflammation by secreting glucorhamnan, which induces tumor necrosis factor-α and disrupts the mucus layer (58, 59). Its enrichment in frail elderly individuals is associated with elevated pro-inflammatory factors and contributes to sarcopenia (60). Ruminococcus abundance is increased in lupus nephritis, coronary heart disease, and CKD, where it induces autoantibodies, promotes lipid deposition, and produces the toxin p-cresyl sulfate, driving immune imbalance, insulin resistance, and cardiovascular injury (58, 61, 62). In this study, its abundance increased with proteinuria severity, suggesting involvement in the progression of proteinuria and renal impairment through intestinal inflammation and metabolite accumulation. Collectively, these pathogenic bacteria interact with the gut-kidney axis to drive progressive renal function decline and increase the risk of cardiovascular complications.

This study also performed a metabolic analysis of the gut microbiota, revealing progressive alterations in metabolic functions across the HC, PROU-L, and PROU-M groups. KEGG pathway analysis indicated that, with worsening proteinuria, the nitrogen metabolism pathway (ko00910) of the gut microbiota became progressively overactivated, and the unsaturated fatty acid synthesis pathway (ko01040) was inhibited. Key enzymes, such as fumarate reductase (EC:1.3.4.1), were significantly correlated with the degree of proteinuria. These functional changes suggest that gut microbiota dysbiosis contributes to CKD progression by promoting uremic toxin production and diminishing anti-inflammatory capacity, providing novel insights into the pathological mechanisms of the gut-kidney axis in CKD patients with varying proteinuria levels and establishing a basis for targeted microecological interventions. Previous studies have shown that gut microbiota can generate polyunsaturated fatty acid (PUFA) precursors through the metabolism of dietary lipids or directly participate in the biotransformation of unsaturated fatty acids, with key enzymes involved in unsaturated fatty acid synthesis (ko01040) playing a crucial role (63). In CKD, dysbiosis may reduce the abundance of PUFA-producing microbiota. In this study, the distribution of enzymes involved in unsaturated fatty acid synthesis (ko01040) progressively declined from the HC group to the PROU-L and PROU-M groups, potentially leading to insufficient PUFA synthesis and thereby exacerbating hypertriglyceridemia and impairing HDL function (64). Supplementation with ω-3 PUFAs can improve the lipid profile in CKD patients, reduce inflammatory mediators, such as C-reactive protein, attenuate pro-fibrotic factors, including Monocyte Chemoattractant Protein-1, and inhibit macrophage infiltration and renal fibrosis (52). Additionally, ω-3 PUFAs enhance endothelial function by activating endothelial nitric oxide synthase and may reduce the risk of cardiovascular complications (64, 65). Dysregulation of enzymes involved in unsaturated fatty acid synthesis (ko01040) may therefore exacerbate CKD progression across proteinuria stages via pro-inflammatory, lipotoxic, and oxidative stress pathways. Targeting the microbiota-PUFA metabolic axis, for instance, through probiotics or ω-3 supplementation, represents a potential strategy to mitigate proteinuria and provide precision intervention. Fumarate reductase (FRD), primarily encoded by Enterococcus species, such as Enterococcus faecalis and Enterococcus faecium, catalyzes the reduction of fumarate to succinate under anaerobic conditions, thereby promoting propionic acid production (66, 67). Propionic acid serves as a substrate for hepatic gluconeogenesis and improves energy metabolism (68). SCFAs also reduce renal inflammation and oxidative stress by inhibiting HDAC and modulating Treg cell differentiation (69). A decrease in FRD-active bacteria may result in SCFA deficiency, exacerbate intestinal barrier dysfunction, increase uremic toxin accumulation, and thereby accelerate the progression of proteinuria.

Nitrogen metabolism is closely associated with gut microbiota dysbiosis in CKD patients with varying grades of proteinuria. The kidney is the central organ for nitrogen metabolism, maintaining nitrogen homeostasis through the excretion of urea and ammonia, while the gut microbiota can modulate nitrogen balance via pathways, including ammonia metabolism, urea hydrolysis, and amino acid deamination (70). In CKD, microbial imbalance leads to abnormal abundance of ammonia-producing bacteria and altered activity of ammonia-assimilating enzymes, resulting in disruptions of ammonia metabolism pathways (71). The progressive activation of the nitrogen metabolism pathway (ko00910) across the HC, PROU-L, and PROU-M groups may generate excessive ammonia, which damages the intestinal barrier, promotes systemic ammonia accumulation, induces inflammatory responses, and accelerates glomerulosclerosis and tubular injury (12). Circulating ammonia levels have been reported to correlate significantly with the degree of renal function decline (72). IgA-specific serine endopeptidase (IgA protease) is also closely linked to gut microbiota dysbiosis in CKD patients with varying proteinuria grades. These enzymes are secreted primarily by pathogenic bacteria, including Haemophilus influenzae and Streptococcus pneumoniae, as well as symbiotic species such as Clostridium ramosum. They specifically cleave the hinge region of IgA1, compromising mucosal immune defense (73). In this study, IgA protease was positively correlated with Haemophilus and was significantly enriched in the PROU-M group, increasing the production of pathogenic galactose-deficient IgA1 (Gd-IgA1). Elevated Gd-IgA1 forms immune complexes with autoantibodies, which deposit in the glomerular mesangium, activate the complement system (e.g., C3 and lectin pathways), induce local inflammation, oxidative stress, and podocyte injury, and directly contribute to proteinuria progression (74). For example, administration of symbiont-derived IgA protease (such as AK183) has been shown in murine models to degrade abnormal IgA deposits, reducing mesangial deposition and proteinuria (73, 75). These findings suggest that modulating the balance of IgA protease-producing microbiota or targeting enzyme activity may offer a novel strategy to delay CKD progression across different proteinuria grades.

The limitations of this study include its single-center, cross-sectional design and limited sample size, which may introduce selection bias and preclude the establishment of direct causal relationships between microbiota and proteinuria. Future investigations should involve multi-center prospective cohorts, animal models, or fecal microbiota transplantation for validation. Functional predictions of gut microbiota based on 16S rRNA sequencing via PICRUSt2 also have inherent limitations, relying on the annotation of known microbial genomes in public databases. Consequently, predictions may not accurately represent the functions of uncharacterized or newly discovered species, as they are inferred from phylogenetic relationships and gene copy number estimation rather than actual transcriptional or translational activity. Post-transcriptional modifications and environmental regulation of functional expression are not accounted for, and the absence of direct metabolomic data necessitates validation through measurement of microbial metabolites in future studies. Additionally, information on diet (e.g., dietary fiber intake) and medications was not collected, which may confound microbiota findings. Subsequent studies should include standardized dietary and medication records and employ advanced sequencing technologies.

In this study, gut microbiota of CKD patients exhibited progressive dysbiosis with increasing proteinuria (from HC to PROU-L to PROU-M): beneficial genera (such as Akkermansia and Coprococcus) decreased, pathogenic genera (such as Enterobacter and Haemophilus) increased, and overall microbial diversity declined. Enterotype transformation was also observed: the healthy group was dominated by the Faecalibacterium-dominated Type 3 enterotype, whereas the PROU-M group shifted to the Prevotella-dominated Type 1 enterotype. Functional abnormalities, including enhanced nitrogen metabolism and reduced unsaturated fatty acid synthesis, were identified, and differential bacteria were significantly correlated with clinical indicators, such as renal function and blood pressure. These findings indicate that gut microbiota dysbiosis contributes to CKD progression and proteinuria aggravation, and that probiotics, such as Coprococcus and Akkermansia, may represent targets for early intervention.

## Data Availability

The raw 16S rRNA gene sequencing data and corresponding sample metadata in this study have been deposited in the NCBI Sequence Read Archive (SRA) under BioProject accession number PRJNA1434534.

## SUPPLEMENTAL MATERIAL

Figure S1 spectrum.03294-25-s0001.doc Association between differential gut microbiota and clinical indicators in HC vs. PROU-L and HC vs PROU-M.

Supplemental tables spectrum.03294-25-s0002.xlsx Tables S1 to S18.

ASM does not own the copyrights to Supplemental Material that may be linked to, or accessed through, an article. The authors have granted ASM a non-exclusive, world-wide license to publish the Supplemental Material files. Please contact the corresponding author directly for reuse.
