# Supplementary material for: Linkage of gut microbiota dysbiosis to chronic kidney disease in patients with graded proteinuria levels
Source: Microbiol Spectr. 2026 Apr 23;14(6):e03294-25. doi: 10.1128/spectrum.03294-25 (PMC13228085; doi:10.1128/spectrum.03294-25)
Supplement: Figure S1 — Association between differential gut microbiota and clinical indicators in HC vs. PROU-L and HC vs PROU-M. [file spectrum.03294-25-s0001.doc]

**
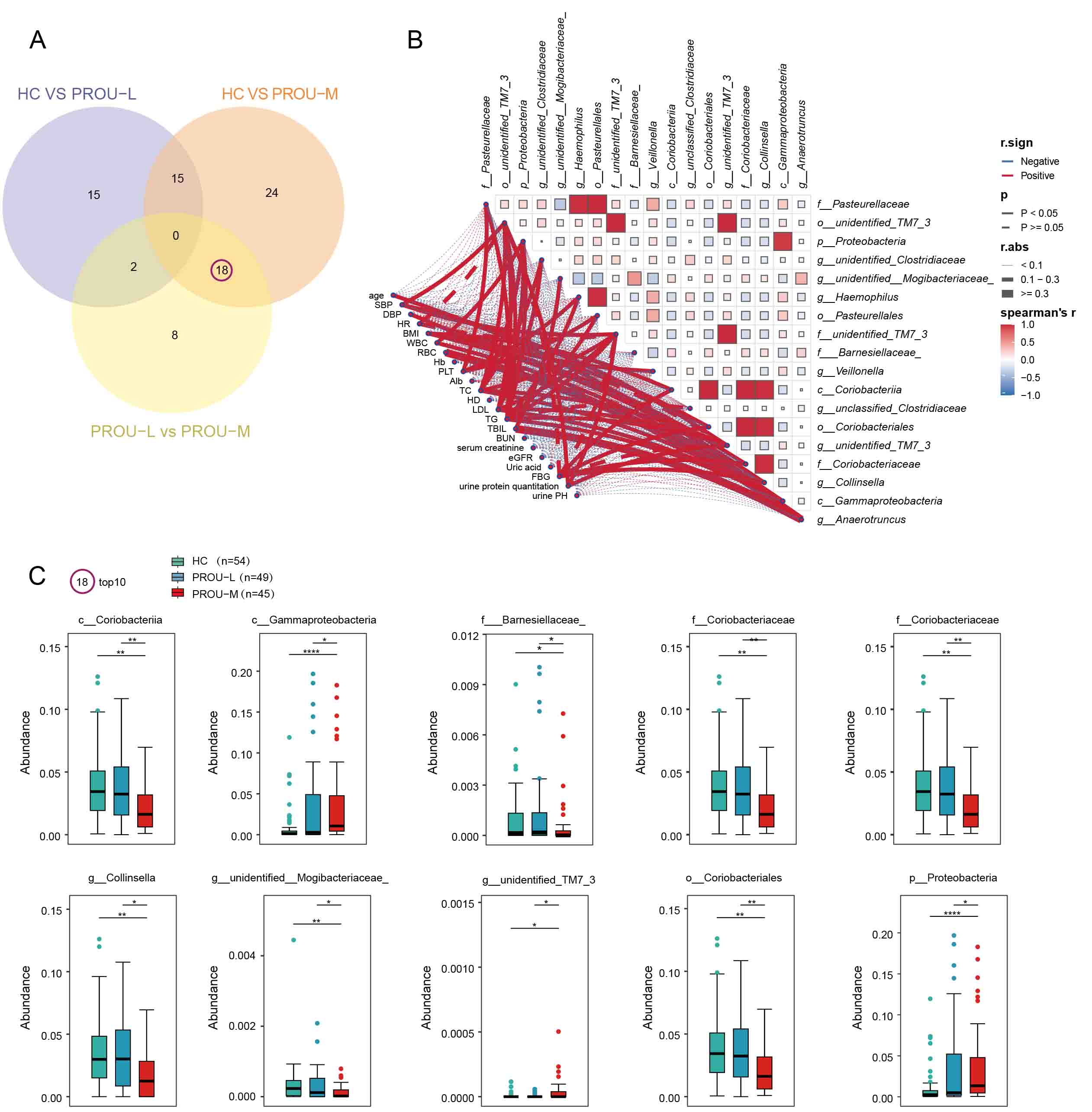
**

**Figure S1. Association between differential gut microbiota and clinical indicators in HC vs. PROU-L and HC vs. PROU-M.** (A) Venn diagram showing the shared and unique differential microbiota among the three group comparisons: HC group vs. PROU-L group; HC group vs. PROU-M group; PROU-L group vs. PROU-M group. (B) Oblique triangular heatmap and network connections showing positive (red connections) and negative (blue connections) correlations between clinical indicators (including gender, age, systolic blood pressure (SBP), diastolic blood pressure (DBP), heart rate (HR), body mass index (BMI), white blood cell count (WBC), red blood cell count (RBC), hemoglobin (Hb), platelet count (PLT), albumin (Alb), total cholesterol (TC), high-density lipoprotein cholesterol (HDL), low-density lipoprotein cholesterol (LDL), triglycerides (TG), aspartate transaminase (AST), alanine transaminase (ALT), total bilirubin (TBIL), blood urea nitrogen (BUN), serum creatinine, estimated glomerular filtration rate (eGFR), uric acid, fasting blood glucose (FBG), urine protein quantitation, urine pH) and gut microbiota (at genus level). The degree of correlation was derived from Spearman correlation analysis (solid lines for P < 0.05, dashed lines for P ≥ 0.05). (C) Abundance distribution of the top 10 of 18 shared differential genera in HC group vs. PROU-M group and PROU-L group vs. PROU-M group among the HC, PROU-L, and PROU-M groups.
